# Serotonin and Social Norms: Tryptophan Depletion Impairs Social Comparison and Leads to Resource Depletion in a Multiplayer Harvesting Game

**DOI:** 10.1177/0956797614527830

**Published:** 2014-05-08

**Authors:** Amy C. Bilderbeck, Gordon D. A. Brown, Judi Read, Mark Woolrich, Phillip J. Cowen, Tim E. J. Behrens, Robert D. Rogers

**Affiliations:** 1 Department of Psychiatry, University of Oxford; 2 Department of Psychology, University of Warwick; 3 Nuffield Department of Primary Healthcare Sciences, University of Oxford; 4 Oxford Centre for Human Brain Activity, University of Oxford; 5 Oxford Centre for Functional MRI of the Brain, University of Oxford; 6 School of Psychology, Bangor University

**Keywords:** social behavior, social influences, neurotransmitters

## Abstract

How do people sustain resources for the benefit of individuals and communities and avoid the tragedy of the commons, in which shared resources become exhausted? In the present study, we examined the role of serotonin activity and social norms in the management of depletable resources. Healthy adults, alongside social partners, completed a multiplayer resource-dilemma game in which they repeatedly harvested from a partially replenishable monetary resource. Dietary tryptophan depletion, leading to reduced serotonin activity, was associated with aggressive harvesting strategies and disrupted use of the social norms given by distributions of other players’ harvests. Tryptophan-depleted participants more frequently exhausted the resource completely and also accumulated fewer rewards than participants who were not tryptophan depleted. Our findings show that rank-based social comparisons are crucial to the management of depletable resources, and that serotonin mediates responses to social norms.

Humans, like other socialized species, must work together to manage the exploitation of valuable but finite resources (Committee on the Human Dimensions of Global Change, 2002; Ostrom, 2009). Supplies such as fresh water, fish, or fossil fuels may form the basis for survival, and a sustainable balance between exploitation and conservation must be found. Game-theory formulations typically assume that such dilemmas pit the self-interested objectives of individuals against the interests of their community (Burke, 2001; Hardin, 1968; Kollock, 1998). Such approaches tend to highlight outcomes illustrating the *tragedy of the commons*, in which resources are eventually exhausted, to the disadvantage of both individuals and their social groups (Hardin, 1968). Field studies, however, demonstrate that community members typically find ways to manage resource-harvesting behaviors through simple coordination, regulation, or sanctions (Feeny, Berkes, McCay, & Acheson, 1990; Ostrom, 2000; Rankin, Bargum, & Kokko, 2007). In the study reported here, we examined the neurochemical mechanisms that mediate how individuals interact with others to manage common-pool resources.

One neurochemical that might modulate the way that individuals solve resource dilemmas is serotonin. Increased serotonin activity—achieved by administration of the amino acid tryptophan in vervet monkeys (Raleigh, McGuire, Brammer, Pollack, & Yuwiler, 1991) and the selective serotonin reuptake inhibitor paroxetine in humans (Knutson et al., 1998)—can facilitate affiliative behaviors. By contrast, reduced serotonin activity (achieved by tryptophan depletion) blocks reciprocal altruism in humans (Wood, Rilling, Sanfey, Bhagwagar, & Rogers, 2006) and facilitates the punishment of unfairness in ways comparable with focal lesions of the ventromedial prefrontal cortex, a cortical region that receives dense serotonergic innervation (Crockett, Clark, Tabibnia, Lieberman, & Robbins, 2008; Koenigs & Tranel, 2007). Reduced serotonin activity also diminishes the reward value of fairness, but enhances the tendency to punish unfairness, via modulated activity within the ventral and dorsal striatum, respectively (Crockett et al., 2013). Taken together, these data suggest that serotonin activity regulates a tension between fairness and norm-enforcing retaliation to influence how people work to meet group-based objectives, such as managing resources.

We tested the effects of diminishing central serotonin activity (through tryptophan depletion; Moore et al., 2000) on the capacity of healthy adults to solve resource dilemmas within small social groups. Participants were introduced to three people (in fact, experimental confederates), and the four-player group was given access to a resource of nominal value (points) that could be harvested and, after the experiment, converted into monetary payoffs. On each harvesting opportunity, group members decided how much to harvest from the resource, which was reduced by the total value of the group’s harvests but then replenished.

Our focus was the role of serotonin in descriptive social norms. Comparisons with social partners’ behavior may be important in resource-management problems (Biel & Thøgersen, 2007; Fischbacher & Gächter, 2010; Thøgersen, 2008), as when people are nudged into reducing their energy consumption by being told that they are using more energy than their neighbors (Nolan, Schultz, Cialdini, Goldstein, & Griskevicius, 2008). People can also be highly sensitive to their ranked position within distributions of behavior (here called *social norms*; Boyce, Brown, & Moore, 2010; Melrose, Brown, & Wood, 2012; Wood, Boyce, Moore, & Brown, 2012). We hypothesized that social ranks would be important when people chose how much to harvest from a shared resource. We tested the hypotheses that people would adjust their current harvests (in an upward or downward direction) on the basis of the (ranked) position of their previous harvests within the social norm represented by the distribution of group members’ harvests, and that these adjustments would be influenced by central serotonin activity.

To vary the social norms, we manipulated the harvesting behavior of the other players (i.e., confederates) at different points in the resource-dilemma game. Sometimes, other players adopted aggressive overharvesting strategies that depleted the resource and that would, if prolonged, have exhausted the resource completely. At other times, other players adopted conservative underharvesting strategies that increased the resource (early in the game) or tended to sustain its value (toward the end of the game). This experimentally induced variation in other people’s harvesting behavior allowed us to assess participants’ use of social norms.

## Method

The experiment was approved by a National Health Service (England) research ethics committee. Participants provided informed consent after reading an information sheet indicating that the experiment would involve making decisions as part of a small group but not specifying all the details of, or the rationale for playing, the multiplayer resource-dilemma game.

### Participants

Rapid dietary tryptophan depletion diminishes central serotonin activity reliably and safely in healthy adult volunteers (Moore et al., 2000). Thirty-two healthy adults were recruited from Oxford University and the local community; 24 were full-time students. All participants followed a low-protein diet for 24 hr before ingesting an amino-acid drink on the morning of the experiment. The drink consumed by 16 of the participants contained tryptophan (the T+ group), and the drink consumed by the other 16 did not contain tryptophan (the T– group; see the Supplemental Material available online for details). Five hours later, participants played the multiplayer resource-dilemma game. The participants, experimenter, and confederates were all unaware of which treatment participants received.

### Procedure

In an initial session, all participants were assessed using a semistructured interview from the *Structured Clinical Interview for DSM-IV-TR Axis I Disorders* (First, Spitzer, Gibbon, & Williams, 2002). Exclusion criteria included the presence or history of serious physical or psychiatric illness, including mood and addictive disorders. Participants completed measures of cognitive ability (Raven’s Standard Progressive Matrices Sets A, B, C, D, & E; Raven, 1996) and impulsivity (Barratt Impulsiveness Scale; Patton, Stanford, & Barratt, 1995).

On the morning of the experiment, participants arrived at the laboratory at 8:30 a.m. and completed self-report measures of state affect using the Positive and Negative Affect Schedule (PANAS; Watson, Clark, & Tellegen, 1988). Blood samples (6 ml) were taken for baseline measurements of plasma tryptophan. Participants then drank the amino-acid drinks over 30 min and occupied themselves with reading or watching television. Five hours later, immediately prior to the start of the resource-dilemma game, follow-up measures of state affect were taken, as well as a second blood sample.

**Fig. 1.**
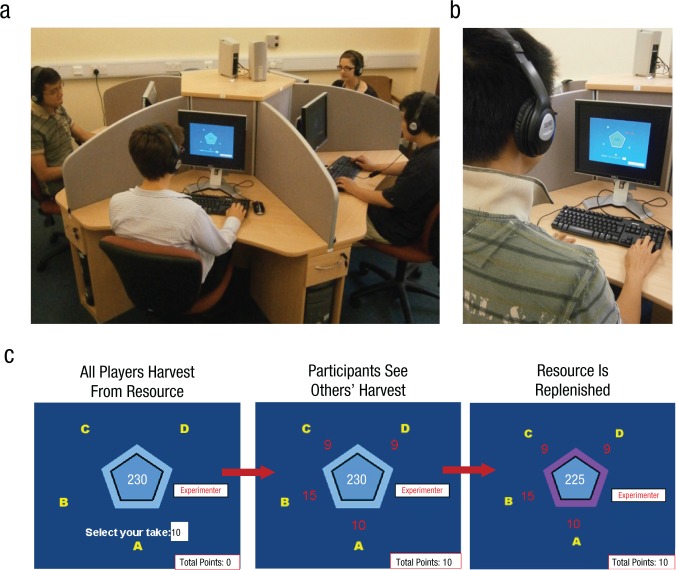
Setup of the multiplayer resource-dilemma game and an example harvesting opportunity. The photographs show (a) the positions of the four players (a participant and three confederates) around the workstation where the game was played and (b) a single participant viewing an example display. In the example harvesting opportunity (c), the letter A indicates the position of the participant at the workstation; the three confederates are represented by B, C, and D, respectively. Throughout the game, the current value of the resource was shown in the center of the pentagon. During the harvesting phase (left panel), participants had 5 s to choose how much to harvest from the resource and enter that amount in the box marked “Select your take:.” This was followed by the 1.5-s observation phase (middle panel), in which the amount each player chose was displayed in red next to his or her identifying letter, and then the participant’s chosen amount was added to his or her total points. The resource was then partially replenished (right panel). Replenishment was signaled by a mauve border appearing around the pentagon and the sounding of a 0.5-s tone.

All four players (i.e., the participant and three confederates) sat at separate workstations around a pentagonal table, with each person’s position identified by labels (A, B, C, and D). The experimenter sat at the fifth workstation (Fig. 1a). Each workstation contained a computer terminal, and dividers concealed other players’ displays and keyboard finger movements (Fig. 1b) but not their faces. First names of players, but no other information, were known among the group before the game began. To enhance the deception that the other three players of the game were genuine, we trained participants alongside one opposite-gender confederate but informed them that the two remaining players (one male, one female) were being trained elsewhere. This procedure avoided both the possibly unhelpful effects of being instructed as part of a larger group (e.g., by making it easier for participants to ask questions) and improved the believability of the game by making participants aware of confederates’ involvement before they took their seats for the game proper. All groups included two male and two female players.

Participants and confederates were given access to a resource, initially of 230 points. On every harvesting opportunity, each player took up to 20 points from the resource. Participants were told that these points, accumulated over the whole game, could be exchanged for a real monetary reward at the end of the experiment (up to a maximum value of £15).

Participants made their harvesting decisions privately, but subsequently observed all other players’ harvests. Participants could see their own (but not others’) total number of accumulated points at all times during the game. Players were explicitly told that the group as a whole, and they as individuals, would make more points if the group were able to find a way to sustain the resource over the course of the game. They were also told that the game would terminate when either the resource was reduced to 0 or an unknown time interval had elapsed. In fact, the game terminated automatically after 109 consecutive harvests.

Figure 1c shows a typical display sequence. Throughout the game, the current value of the resource was shown in the center of a light-blue pentagon. The value participants selected for the current harvest was shown below the pentagon. On each harvesting opportunity, participants were allowed to harvest points by pressing the up-arrow and down-arrow keys of a keyboard to scroll through integer values. (The confederates pretended to harvest points in the same manner, although their harvests were actually decided by the computer.) The harvest was the value selected 5 s after the appearance of the display. Two seconds later, the value of the participant’s harvest was added to his or her accumulated total points. One second after that, all four players’ harvests were displayed in red alongside the letters that indicated their position around the table. The resource was updated 1.5 s later by subtracting the summed value of all harvests, and then the resource was at least partially replenished (see the next paragraph). This event was signaled by changing the pentagon color border to mauve and playing a 0.5-s tone. Harvesting opportunities were separated by 1.5-s intervals.

The replenishment operated as follows. First, the resource was increased by a number of points drawn from a rectangular distribution of integers between 17% and 23% of the resource that remained following all players’ harvests. Second, the value of the replenished resource was weighted by one-third of the previous resource value. This running-total mechanism dampened fluctuations in resource value over the course of the game, increased the number of harvesting opportunities that participants were likely to complete in a game, and facilitated the emergence of stable resource-management strategies (see the Supplemental Material for an example replenishment).

Communication between playing partners was not allowed during the game, and confederates maintained their focus of attention on the game displays throughout. All players were told to press the space bar on their keyboards to start the game. In fact, the computer controlling the experiment responded only to the participants’ button responses. All players wore headphones, which provided sound effects for the game and background music (Mozart concertos) to reduce possible noise distractions.

The over- and underharvesting behavior of confederates instantiated four social environments (see the Supplemental Material for details). In the one-overharvester environment, a single confederate overharvested the resource (e.g., Fig. 1c); in the two-overharvesters environment, two confederates overharvested the resource. By contrast, in the one-underharvester environment, one confederate underharvested the resource, whereas, in the two-underharvesters environment, two confederates underharvested the resource. Overharvesting by confederates was geared (assuming that the harvests of the remaining players had no impact) to diminish the resource, whereas underharvesting behavior was geared to increase the resource. Therefore, the value of the resource available to the players tended to decrease in the overharvesting environments but increase in the underharvesting environments. In each social environment, all other players (i.e., any confederates who were not saliently over- or underharvesting) harvested amounts that, by themselves, placed a gentle downward pressure on the value of the resource. (Extensive pilot studies suggested that healthy adults tend to overharvest from a shared resource, other things being equal, and we wanted participants to be required to manage resources that were under a mild threat of exhaustion.)

There were 13 consecutive harvesting opportunities in each of the four harvesting environments. The four environments were presented in a pseudorandom order over the 109 harvests of the game (up to two repetitions of each environment). One-overharvester environments never immediately preceded or followed two-overharvesters environments; one-underharvester environments never immediately preceded or followed two-underharvesters environments. The first five harvests of the game were warm-ups, in which the harvests of playing partners maintained the resource at roughly its initial level.

After finishing the resource-dilemma game, participants completed a postgame questionnaire to measure their satisfaction with the game and the strategies that they employed. Participants rated three items: “How happy were you with the outcome of the game?” “How much did you feel that you wanted to act in your own benefit when selecting your ‘take’?” and “How much did you feel that you thought of the best outcome for the group when selecting your ‘take’?” Responses were made using 7-point Likert scales, with the anchors *not at all* (0) and *very much* (6) and a midpoint of *somewhat* (3).

## Results

There were no significant differences in the age, cognitive ability, trait affect, or impulsivity of T– and T+ participants (Table 1), −1.26 (*SE* = 1.43) < βs < 4.71 (*SE* = 2.96). As expected (Moore et al., 2000), plasma (total) tryptophan between baseline (immediately before receiving the amino-acid drink) and 5 hr later was reduced following the T– treatment but increased following the T+ treatment (see Table S1 in the Supplemental Material), β = −14.66 (*SE* = 1.26), *p* < .0001. Plasma tryptophan was reduced in the T– compared with the T+ participants just before participants began the resource-dilemma game, β = −14.38 (*SE* = 1.08), *p* < .0001. Consistent with previous findings (Booij et al., 2002; Ruhe, Mason, & Schene, 2007), results showed that the T– treatment did not produce marked changes in state positive or negative affect (Table S1 in the Supplemental Material), −1.88 (*SE* = 3.20) < βs < −0.94 (*SE* = 0.86). All participants reported being deceived about the veracity of the confederates’ behavior, as indicated by scores of 4 or higher on the measure of deception. Deception scores for T+ participants (*M* = 6.71, *SE* = 0.18) and T– participants (*M* = 6.33, *SE* = 0.33) were not significantly different, β = 0.40 (*SE* = 0.41).

**Table 1.** Descriptive Statistics for Participants Who Consumed Amino-Acid Drinks Containing (T+) or Not Containing (T–) Tryptophan

Treatment group	Gender	Mean age (years)	Mean Raven’s matrices score	Mean BIS-11 score	Mean PANAS trait score	
					Positive affect	Negative affect
T+	7 male, 9 female	24.44 (1.65)	55.53 (1.17)	58.00 (2.22)	34.44 (0.96)	13.94 (0.91)
T–	9 male, 7 female	23.56 (0.92)	56.63 (0.80)	62.56 (1.86)	37.00 (1.22)	14.56 (1.06)

Note: Standard errors are given in parentheses. Participants completed measures of cognitive ability (Raven’s Standard Progressive Matrices Sets A, B, C, D, & E; Raven, 1996), impulsivity (Barratt Impulsiveness Scale, or BIS-11; Patton, Stanford, & Barratt, 1995), and positive and negative state affect (Positive and Negative Affect Schedule, or PANAS; Watson, Clark, & Tellegen, 1988).

### Tryptophan depletion and resource-dilemma outcomes

**Fig. 2.**
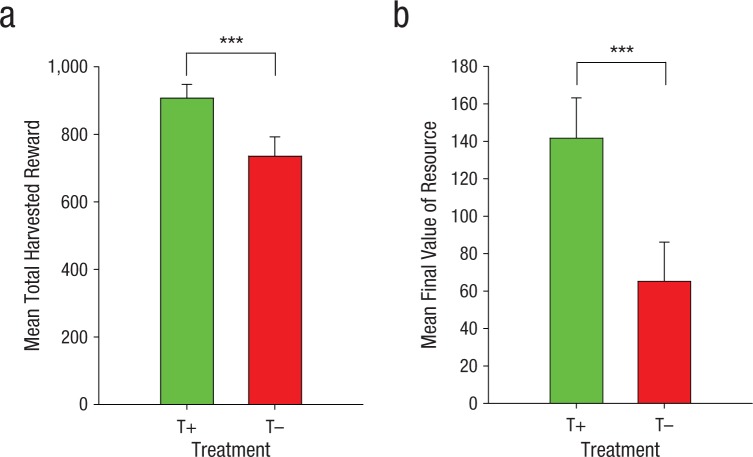
Mean total number of points harvested (a) and mean final resource value (b) at the end of the multiplayer resource-dilemma game as a function of whether participants consumed (T+) or did not consume (T–) a drink containing the serotonin precursor tryptophan. Error bars show standard errors. Asterisks indicate a significant difference between conditions (***p < .005).

First, we compared the outcomes of the resource-dilemma game and the total points harvested by the T– participants and the T+ participants. Eight of the T– participants (50% of the group) exhausted the resource completely, compared with only 2 of the T+ participants (12.5%), χ^2^(1, *N* = 32) = 5.24, *p* = .022. The T– participants also accumulated fewer points by the end of the game (Fig. 2a), β = −294.06 (*SE* = 79.39), *p* = .0002, and finished the game with smaller final resource values than the T+ participants (see Fig. 2b), β = −122.42 (*SE* = 43.06), *p* = .0045.

Thus, reductions in serotonin activity, achieved by tryptophan depletion, induced a bias toward aggressive harvesting behaviors that, in extremis, exhausted the shared resource completely. Overall, there was little evidence that the resource outcomes were consistently related to total or subscale scores of motor, attentional, and nonplanning impulsivity in either treatment group (–.25 < *r*s < .44); however, T– participants with higher nonplanning impulsivity scores did tend to accumulate larger totals of reward by the end of the game (*r* = .57, *p* = .02, uncorrected for the eight Pearson’s correlation tests conducted).

### Tryptophan depletion and sensitivity to social norms

**Fig. 3.**
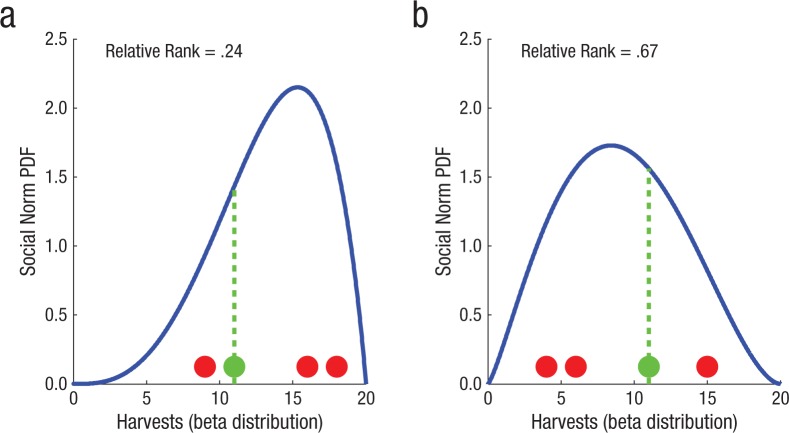
Illustration of descriptive social norms: (a) overharvesting and (b) underharvesting social environments of the multiplayer resource-dilemma game. Each panel shows four harvests (dots) and a probability density function (PDF; beta distributions scaled to between 0 and 20) representing the corresponding social norms. Relative rank is the position of a harvest of 12 (green dot) in the cumulative PDFs of the two different social norms. The vertical lines highlight the lower ranking of a harvest of 12 in the (a) overharvesting environment than in the (b) underharvesting environment.

To explore the effects of social norms further, we constructed regression models of the structural (and social) features of the game that influenced how participants adjusted their harvests from one harvesting opportunity to the next (Table 2; see the Supplemental Material for further details). Regressors included terms to code (a) the value of participants’ own immediately preceding harvests; (b) the current value of the available resource; (c) the presence of one or two other players who were overharvesting, modeled as a binary indicator (with the presence of one or two other players who were underharvesting as the referent); (d) the rank of participants’ last harvest within the distribution of all four players’ last harvests (i.e., their position within the social norms; see Fig. 3); and (e) the T– treatment, modeled as a binary indicator with the T+ treatment as the referent. (Statistical significance was tested against a threshold of *p* < .05; bootstrapped models that provided 95% confidence intervals for the coefficients described below can be found in Table S2 in the Supplemental Material.)

**Table 2.** Results of the Regression Analysis Predicting Participants’ Adjustments in Their Harvests

Predictor	Model 1		Model 2	
	T+ treatment only	T+ treatment vs. T– treatment	T+ treatment only	T+ treatment vs. T– treatment
Intercept	0.75 (0.26)**	—	0.79 (0.53)	—
Treatment group	—	−0.29 (0.38)	—	−0.71 (0.56)
Value of participant’s own immediately preceding harvest	−0.69 (0.03)****	0.07 (0.03)*	−0.66 (0.04)****	—
Current value of available resource	0.02 (0.001)****	0.001 (0.002)	0.03 (0.002)	—
Presence of one or two other players who were overharvesting	0.88 (0.15)****	−0.39 (0.22)	0.80 (0.20)****	—
Rank of participants’ last harvest within the distribution of all four players’ last harvests (Rank_*n*−1_)	—	—	−6.56 (3.33)*	10.14 (4.66)*
(Rank_*n*−1_)^2^	—	—	18.28 (7.35)*	−23.73 (10.39)*
(Rank_*n*−1_)^3^	—	—	−13.11 (4.83)**	15.66 (6.68)*

Note: Standardized regression coefficients are shown, and standard errors are given in parentheses. For the group that consumed the amino-acid drink containing tryptophan (T+; *n* = 16), there were 1,569 observations; for the group that consumed the amino-acid drink that did not contain tryptophan (T–; *n* = 16), there were 1,217 observations.

* *p* < .05. **p < .01. ****p < .0001.

Model 1 showed that T+ participants tended to harvest more from higher value resources than from lower value resources (Table 2), β = 0.02 (*SE* = 0.001), *p* < .0001, and this behavior was not markedly altered in the T– compared with the T+ participants, β = 0.001 (*SE* = 0.002). However, tryptophan depletion did diminish the conditionality of cooperation seen in other common-pool problems (Fischbacher, Gächter, & Fehr, 2001). Specifically, the T+ participants tended to reduce their harvests when they had taken larger harvests on the preceding harvesting opportunity, β = −0.69 (*SE* = 0.03), *p* < .0001, but then tended to increase their harvests when one or two other players were overharvesting, β = 0.88 (*SE* = 0.15), *p* < .0001. These behaviors indicate that the T+ participants’ willingness to moderate their own harvesting was, in general, limited by the aggressive harvesting of other players. By contrast, the tendency to reduce, or self-correct, harvests immediately following larger harvests was attenuated in the T– participants, β = 0.07 (*SE* = 0.03), *p* = .02. These observations, together with the fact that a greater number of T– compared with T+ participants exhausted the resource completely, indicates that temporary reduction of serotonin activity induces aggressive but less flexible harvesting strategies when people manage valuable resources.

Of particular interest was whether participants’ harvests were sensitive to the ranked position of their own harvest amounts within the social norms and whether this sensitivity can be disrupted by temporary disruptions of serotonergic neuromodulation. We hypothesized that participants used the value of all four players’ previous harvests to estimate the social norm currently operating within the resource-dilemma game. These social norms were modeled as beta distributions; that is, we fitted beta distributions, scaled between 0 and 20, to each set of four harvests from each harvesting opportunity, and we used that distribution to represent the social norm. Figure 3 illustrates this process in an overharvesting (Fig. 3a) and an underharvesting (Fig. 3b) environment (see also Fig. S1 in the Supplemental Material).

**Fig. 4.**
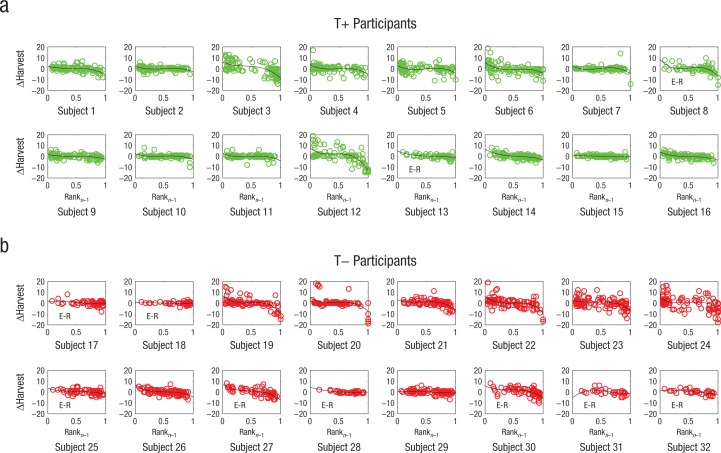
Adjustments in harvests from one harvesting opportunity to the next (ΔHarvest) as a function of the rank of participants’ last harvest within the beta distributions of all four players’ last harvests (i.e., their position within the social norms; Rank_*n*–1_). Results are shown separately for each of the 16 participants in each treatment group. Lines represent best-fitting third-order polynomials; the “E-R” notation identifies participants who exhausted the resource.

We found exactly this predicted pattern. Figure 4 shows the adjustment in participants’ harvests from one harvesting opportunity to the next, ΔHarvest, as a function of the rank of their last harvest, separately for each participant. Our prediction was clearly confirmed in the behavior of the majority of the T+ participants. Moreover, Model 2 showed that the relationship between the two factors tended to be markedly nonlinear, as indicated by significant regression coefficients for the three elements of a polynomial (Table 2), β = −6.56 (*SE* = 3.33), β = 18.28 (*SE* = 7.35), β = −13.11 (*SE* = 4.83), all *p*s < .05. The T+ participants made the largest upward and downward adjustments to their harvests when the value of their last harvest fell toward the bottom or top of the distributions of other players’ harvests, respectively. By comparison, the T– participants’ harvests tended to rank toward the top of these distributions and showed only inconsistent use of the social norms in adjusting subsequent harvests (Table 2), β = 10.14 (*SE* = 4.66), β = −23.73 (*SE* = 10.39), β = 15.66 (*SE* = 6.68), all *p*s < .03.

### Effects of treatment on motor responding

Treatment-related changes in resource management or in the sensitivity to social comparisons following tryptophan depletion could not be attributed to heightened motor impulsivity. Neither the speed of the first harvesting response (T– participants: *M* = 0.20 s, *SE* = 0.046; T+ participants: *M* = 0.22 s, *SE* = 0.05) nor the number of button presses per harvest opportunity (T– participants: *M* = 2.89, *SE* = 0.21; T+ participants: *M* = 2.75, *SE* = 0.25) were significantly altered as a function of treatment condition, −0.04 (*SE* = 0.06) < βs *<* 0.09 (*SE* = 0.320).

### Postgame self-report of motivations and objectives

Finally, participants who received the T– treatment were equally happy with the outcome of the resource-dilemma game (*M* = 4.31, *SE* = 0.24), compared with those who received the T+ treatment (*M* = 4.25, *SE* = 0.25), β = −0.14 (*SE* = 0.34). Similarly, the two treatment groups reported acting for their own benefit to a similar extent (T– participants: *M* = 4.25, *SE* = 0.27; T+ participants: *M* = 3.56, *SE* = 0.36), β = 0.55 (*SE* = 0.41). However, the T– participants did report thinking less about the best outcome for the group when completing the game (*M* = 2.75, *SE* = 0.34) than the T+ participants did (*M* = 4.00, *SE* = 0.41), β = −1.11 (*SE* = 0.50), *p <* .05.

## Discussion

In the present experiment, we used a laboratory multiplayer resource-dilemma game and tryptophan depletion to demonstrate that reduced central serotonin activity compromises people’s sensitivity to descriptive social norms while they are managing valuable but depletable resources within small social groups. Individuals who received the T– treatment tended to deplete the shared resources to a significantly greater extent, and accumulate fewer rewards over the course of the game, than individuals who received the T+ treatment. At its most dramatic, this behavior involved aggressive overharvesting that exhausted groups’ resources completely.

We tested the hypothesis that people use their own and others’ harvesting behavior to construct distributions that specify the social norms within resource dilemmas and then use the ranks of their own harvests within these distributions to guide decisions about how much to take from the resource. Our data support this hypothesis: T+ participants increased the amount they harvested when the value of their last harvest was ranked toward the lower tail of these distributions, but decreased the amount they harvested when the value of their last harvest was ranked toward the upper tail. The use of rank-based social norms, however, tended to be disrupted in the T– participants, whose harvests clustered at high ranks toward the upper end of the estimated distributions of other peoples’ harvests, with only inconsistent (downward) adjustments in their subsequent harvests. Our results do not reflect changes in state affect or motor impulsivity following tryptophan depletion (Moore et al., 2000). Thus, to the best of our knowledge, these data constitute the first evidence of a neurochemical—specifically, serotonergic—mechanism underpinning resource management in human groups.

Converging evidence that tryptophan depletion suppressed the regulation of harvesting through diminished sensitivity to social norms is provided by the observation that, whereas the T– and T+ participants were equally happy with the outcomes of their resource-dilemma games and reported acting for their own benefit to a very similar extent, the T– participants reported significantly less concern about the best outcome for the wider group of players than the T+ participants. This suggests that their relatively poor performance, in being more likely to exhaust the resource completely, was paralleled by a reduction in the evaluated worth of the group objective of managing the resource effectively for the collective good.

Further research will be needed to identify the precise psychological mechanisms modulated by serotonin to influence resource management. Our data provided only very limited evidence that tryptophan depletion altered resource-management outcomes to a greater extent in participants with higher psychometric scores of impulsivity. However, the tendency to overharvest following tryptophan depletion may still reflect enhanced temporal discounting (Schweighofer et al., 2008; Tanaka et al., 2007), leading to preferences for larger immediate harvests at the expense of preserving the resources for the long term. Overharvesting in the T– participants, irrespective of social norms, may also be linked to the broader aggressive behaviors sometimes observed following tryptophan depletion, although such effects are strongest in samples specifically selected for high-trait aggression (Dougherty, Bjork, Marsh, & Moeller, 1999). Finally, our sample consisted predominantly of students (both domestic and international), which raises the possibility that their behavior in our resource-dilemma game (following the T+ or T– treatments) was conditioned by cultural factors (Zhu, Gigerenzer, & Huangfu, 2013).

Our laboratory multiplayer resource-dilemma game was also only a very approximate model of real-world resource dilemmas, as it involved no immediate survival or economic imperatives (Committee on the Human Dimensions of Global Change, 2002; Gardiner, 2001; Ostrom, 2009) or sanctions known to promote socially cooperative behavior in common-pool problems (Fehr & Gächter, 2002). Our game also afforded complete information about the harvesting behavior of other players, which allowed participants to estimate social norms accurately. In real-world resource dilemmas, the value of resources harvested by social partners can be uncertain, which can potentially increase the salience of other sources of information—such as fluctuations in the resource value over time—in decisions about harvesting strategies (Jager, Janssen, & Vlek, 2002). However, as in previous experiments (Barclay, 2004; Campbell, Bush, Brunell, & Shelton, 2005), our game was intended to model only some of the psychosocial processes—in this instance, the social norms—that influence the way that people cope with real-life resource-management problems and to demonstrate how these processes are modulated by serotonergic activity.

In summary, notwithstanding the preceding limitations, our findings indicate that serotonin activity is implicated in the ability of human adults to use social norms to manage, as part of an interdependent group, finite but valuable resources.

## References

[bibr1-0956797614527830] Barclay P. (2004). Trustworthiness and competitive altruism can also solve the “tragedy of the commons.” Evolution and Human Behavior, 25, 209–220.

[bibr2-0956797614527830] Biel A. Thøgersen J. (2007). Activation of social norms in social dilemmas: A review of the evidence and reflections on the implications for environmental behaviour. Journal of Economic Psychology, 28, 93–112.

[bibr3-0956797614527830] Booij L. Van der Does W. Benkelfat C. Bremner J. D. Cowen P. J. Fava M. . . . Van der Kloot W. A. (2002). Predictors of mood response to acute tryptophan depletion: A reanalysis. Neuropsychopharmacology, 27, 852–861. 12431859 10.1016/S0893-133X(02)00361-5

[bibr4-0956797614527830] Boyce C. J. Brown G. D. A. Moore S. C. (2010). Money and happiness: Rank of income, not income, affects life satisfaction. Psychological Science, 21, 471–475. 20424085 10.1177/0956797610362671

[bibr5-0956797614527830] Burke B. E. (2001). Hardin revisited: A critical look at perception and the logic of the commons. Human Ecology, 29, 449–476.

[bibr6-0956797614527830] Campbell W. K. Bush C. P. Brunell A. B. Shelton J. (2005). Understanding the social costs of narcissism: The case of the tragedy of the commons. Personality and Social Psychology Bulletin, 31, 1358–1368. 16143668 10.1177/0146167205274855

[bibr7-0956797614527830] Committee on the Human Dimensions of Global Change. (2002). The drama of the commons (Ostrom E. Dietz T. Dolsak N. Stern P. C., Eds.). Washington, DC: National Academies Press.

[bibr8-0956797614527830] Crockett M. J. Apergis-Schoute A. Herrmann B. Lieberman M. Müller U. Robbins T. W. Clark L. (2013). Serotonin modulates striatal responses to fairness and retaliation in humans. Journal of Neuroscience, 33, 3505–3513. 23426678 10.1523/JNEUROSCI.2761-12.2013 PMC3593678

[bibr9-0956797614527830] Crockett M. J. Clark L. Tabibnia G. Lieberman M. D. Robbins T. W. (2008). Serotonin modulates behavioral reactions to unfairness. Science, 320, 1739. 10.1126/science.1155577 PMC2504725 18535210

[bibr10-0956797614527830] Dougherty D. M. Bjork J. M. Marsh D. M. Moeller F. G. (1999). Influence of trait hostility on tryptophan depletion-induced laboratory aggression. Psychiatry Research, 88, 227–232. 10622343 10.1016/s0165-1781(99)00088-8

[bibr11-0956797614527830] Feeny D. Berkes F. McCay B. J. Acheson J. M. (1990). The tragedy of the commons: Twenty-two years later. Human Ecology, 18, 1–19. 12316894 10.1007/BF00889070

[bibr12-0956797614527830] Fehr E. Gächter S. (2002). Altruistic punishment in humans. Nature, 415, 137–140. 11805825 10.1038/415137a

[bibr13-0956797614527830] First M. B. Spitzer R. L. Gibbon M. Williams J. B. W. (2002). Structured Clinical Interview for DSM-IV-TR Axis I Disorders—Patient edition (research version). New York, NY: Biometrics Research.

[bibr14-0956797614527830] Fischbacher U. Gächter S. (2010). Social preferences, beliefs, and the dynamics of free riding in public good experiments. American Economic Review, 100, 541–556.

[bibr15-0956797614527830] Fischbacher U. Gächter S. Fehr E. (2001). Are people conditionally cooperative? Evidence from a public goods experiment. Economics Letters, 71, 397–404.

[bibr16-0956797614527830] Gardiner S. M. (2001). The real tragedy of the commons. Philosophy & Public Affairs, 30, 387–416. 12653120 10.1111/j.1088-4963.2001.00387.x

[bibr17-0956797614527830] Hardin G. (1968). The tragedy of the commons. Science, 162, 1243–1248. 5699198

[bibr18-0956797614527830] Jager W. Janssen M. A. Vlek C. A. J. (2002). How uncertainty stimulates over-harvesting in a resource dilemma: Three possible explanations. Journal of Environmental Psychology, 22, 247–263.

[bibr19-0956797614527830] Knutson B. Wolkowitz O. M. Cole S. W. Chan T. Moore E. A. Johnson R. C. . . . Reus V. I. (1998). Selective alteration of personality and social behavior by serotonergic intervention. American Journal of Psychiatry, 155, 373–379. 9501748 10.1176/ajp.155.3.373

[bibr20-0956797614527830] Koenigs M. Tranel D. (2007). Irrational economic decision-making after ventromedial prefrontal damage: Evidence from the ultimatum game. Journal of Neuroscience, 27, 951–956. 17251437 10.1523/JNEUROSCI.4606-06.2007 PMC2490711

[bibr21-0956797614527830] Kollock P. (1998). Social dilemmas: The anatomy of cooperation. Annual Review of Sociology, 24, 183–214.

[bibr22-0956797614527830] Melrose K. L. Brown G. D. A. Wood A. M. (2012). Am I abnormal? Relative rank and social norm effects in judgments of anxiety and depression symptom severity. Journal of Behavioral Decision Making, 26, 174–184.

[bibr23-0956797614527830] Moore P. Landolt H. P. Seifritz E. Clark C. Bhatti T. Kelsoe J. . . . Gillin J. C. (2000). Clinical and physiological consequences of rapid tryptophan depletion. Neuropsychopharmacology, 23, 601–622. 11063917 10.1016/S0893-133X(00)00161-5

[bibr24-0956797614527830] Nolan J. M. Schultz P. W. Cialdini R. B. Goldstein N. J. Griskevicius V. (2008). Normative social influence is underdetected. Personality and Social Psychology Bulletin, 34, 913–923. 18550863 10.1177/0146167208316691

[bibr25-0956797614527830] Ostrom E. (2000). Reformulating the commons. Swiss Political Science Review, 6(1), 29–52.

[bibr26-0956797614527830] Ostrom E. (2009). A general framework for analyzing sustainability of social-ecological systems. Science, 325, 419–422. 19628857 10.1126/science.1172133

[bibr27-0956797614527830] Patton J. H. Stanford M. S. Barratt E. S. (1995). Factor structure of the Barratt Impulsiveness Scale. Journal of Clinical Psychology, 51, 768–774. 8778124 10.1002/1097-4679(199511)51:6<768::aid-jclp2270510607>3.0.co;2-1

[bibr28-0956797614527830] Raleigh M. J. McGuire M. T. Brammer G. L. Pollack D. B. Yuwiler A. (1991). Serotonergic mechanisms promote dominance acquisition in adult male vervet monkeys. Brain Research, 559, 181–190. 1794096 10.1016/0006-8993(91)90001-c

[bibr29-0956797614527830] Rankin D. J. Bargum K. Kokko H. (2007). The tragedy of the commons in evolutionary biology. Trends in Ecology & Evolution, 22, 643–651. 17981363 10.1016/j.tree.2007.07.009

[bibr30-0956797614527830] Raven J. (1996). Standard progressive matrices sets A, B, C, D & E. Oxford, England: Oxford Psychologists Press.

[bibr31-0956797614527830] Ruhe H. G. Mason N. S. Schene A. H. (2007). Mood is indirectly related to serotonin, norepinephrine and dopamine levels in humans: A meta-analysis of monoamine depletion studies. Molecular Psychiatry, 12, 331–359. 17389902 10.1038/sj.mp.4001949

[bibr32-0956797614527830] Schweighofer N. Bertin M. Shishida K. Okamoto Y. Tanaka S. C. Yamawaki S. Doya K. (2008). Low-serotonin levels increase delayed reward discounting in humans. Journal of Neuroscience, 28, 4528–4532. 18434531 10.1523/JNEUROSCI.4982-07.2008 PMC6670945

[bibr33-0956797614527830] Tanaka S. C. Schweighofer N. Asahi S. Shishida K. Okamoto Y. Yamawaki S. Doya K. (2007). Serotonin differentially regulates short- and long-term prediction of rewards in the ventral and dorsal striatum. PLoS ONE, 2(12), Article e1333. Retrieved from http://www.plosone.org/article/info%3Adoi%2F10.1371%2Fjournal.pone.0001333 10.1371/journal.pone.0001333 PMC2129114 18091999

[bibr34-0956797614527830] Thøgersen J. (2008). Social norms and cooperation in real-life social dilemmas. Journal of Economic Psychology, 29, 458–472.

[bibr35-0956797614527830] Watson D. Clark L. A. Tellegen A. (1988). Development and validation of brief measures of positive and negative affect: The PANAS scales. Journal of Personality and Social Psychology, 54, 1063–1070. 3397865 10.1037//0022-3514.54.6.1063

[bibr36-0956797614527830] Wood A. M. Boyce C. J. Moore S. C. Brown G. D. A. (2012). An evolutionary based social rank explanation of why low income predicts mental distress: A 17 year cohort study of 30,000 people. Journal of Affective Disorders, 136, 882–888. 22078299 10.1016/j.jad.2011.09.014

[bibr37-0956797614527830] Wood R. M. Rilling J. K. Sanfey A. G. Bhagwagar Z. Rogers R. D. (2006). Effects of tryptophan depletion on the performance of an iterated prisoner’s dilemma game in healthy adults. Neuropsychopharmacology, 31, 1075–1084. 16407905 10.1038/sj.npp.1300932

[bibr38-0956797614527830] Zhu L. Gigerenzer G. Huangfu G. (2013). Psychological traces of China’s socio-economic reforms in the ultimatum and dictator games. PLoS ONE, 8(8), Article e70769. Retrieved from http://www.plosone.org/article/info%3Adoi%2F10.1371%2Fjournal.pone.0070769 10.1371/journal.pone.0070769 PMC3743878 23967102

